# Supplementary material for: Food insecurity and water management shocks in Saudi Arabia: Bayesian VAR analysis
Source: PLoS One. 2024 Jan 24;19(1):e0296721. doi: 10.1371/journal.pone.0296721 (PMC10807787; doi:10.1371/journal.pone.0296721)
Supplement: Attachment — Submitted filename: Response to Reviewers.docx [file pone.0296721.s002.docx]

PONE-D-23-19746: Food Insecurity and Water Management Shocks in Saudi Arabia: Bayesian VAR Analysis

Dear Editor Journal of PLOS One.

First thanks for all the suggestions and spotting points. Our answers with details in red color after each suggestion in this file. In the main text, we responded to all suggestions with track and red color.

Reviewer #1: Introduction

-First paragraph- spelling- macr-level- should be Macro-

Answer: Corrected to Macro in line 133.

- second paragraph- " exchange rates(EXR)- The exchange rate is the value of one currency relative to another currency. In the context you provided, it is unclear which currency or currencies are being referred to.

Answer: the exchange rate is clarified in line 57 also we insert the footnote for more information.

- After Figure 1 - Note: explained equations - I’m not sure what these equations mean? The authors need to explain with a foot note if possible.

Answer: The equations were explained as Foote notes in lines 66-67.

-"The contribution of this paper. " need to explore in detailed - need to explain "why the paper deserve to publish?' what is the contribution to knowledge - need to explain in detailed with the alignment of literature- what is done ( by referring recent studies up to 12)- then find the gap- explain - the contribution of the knowledge or science explicitly.

Answer: The contribution of the paper was explored in detail with the alignment of literature in lines 89-125.

-The introduction provides overview of the topic of food insecurity and its various factors, as well as the need for research in the context of Saudi Arabia. However, there are a few recommendations to improve the clarity and structure of the introduction: Clear research objective: While the introduction mentions the aim of identifying and estimating the Food Insecurity Multidimensional Index (FIMI) and examining the dynamic effect of sustainable water on food insecurity, it would be helpful to explicitly state the research objective in a clear and concise manner. For example, you could specify whether the objective is to assess the impact of water efficiency on food insecurity in Saudi Arabia or to analyze the factors influencing FIMI in the Middle East.

Answer: We added the objective of the study: to assess the impact of water efficiency on food insecurity in Saudi Arabia in lines 85-86.

Date and Models

-It will be useful to mention which statistical software was used to conduct the analysis in
- page 8- The article does not explicitly mention whether the author considered the possibility of multiple structural breaks in their data. If the author did not consider the possibility of multiple structural breaks in the variables, it should be mentioned as a limitation of the study. The absence of testing for multiple structural breaks could be considered a limitation of the study. Ignoring the possibility of multiple breaks may lead to biased parameter estimates. Clemente-montanes-reyes unit root test is a test capable of testing for two structural breaks (for future reference).

It will be useful to mention which statistical software was used to conduct the analysis in
- page 8-

Answer: Statistical software was mentioned in line 210.

The absence of testing for multiple structural breaks could be considered a limitation of the study.

Answer: We analyzed the multiple structural breaks and the results are included in Table (2) in red color.

-Page 9- Equations 5-6- Just keep in mind that the three models test for the source of non-stationarity. For example if the source of non-stationary is due to a deterministic trend, then one should detrend the data and not difference the data. Differencing a deterministic trend does not make the data stationary but instead introduces a unit root, which makes the problem even worse. To address a deterministic trend, other techniques can be employed, such as detrending the data using regression analysis. Therefore, on should be diligent in identifying the source of non-stationarity in the data since it will determine which transformation will be applied to address non-stationarity. Based on the aforementioned I would advise the authors to make sure that model C is the correct model to be applied.

Answer: Yes, you’re perfect. Trend-stationarity would imply no unit root for the detrended series. Model C has solved these mentioned problems. Because it depends on also on constants. And for that, we accept the VAR and Bayesian VAR models. Equation (8) in line 281. Also, Bayesian VAR models are more flexible than traditional VAR and can handle both stationary and non-stationary data.

Results and discussion
-page 25- (Forecast-error variance decompositions)- This paper do not have a discussion therefore I expect to see in the results somewhere references included that interact with the study’s results. The authors should for example see if they can find studies that have illustrated/found similarly that water use efficiency management for agriculture and industrial purposes reduces food insecurity while increasing water for services use increases food insecurity.

Answer: References for confirmation were added in lines 499-502.

Reviewer #2: The authors have done a good analysis but the paper is not organized appropriately.

Answer: We organized the paper.

English usage in the paper needs to be improved as the paper many sections / sentences in the paper are difficult to be read and understood.

Answer: The article's English usage was revised carefully with a track.

The conclusions of the paper are not properly drawn and presented based on the analysis. The conclusion is mainly repetitions of what is explained in previous sections.

Answer: We rewritten the conclusion section.

The abstract needs to be rewritten to highlight the major findings based on the analysis done.

Answer: The abstract was rewritten.

The paper includes many Tables and Figures. The authors may try to combine those or reduce the number of Tables / Figures.

Answer: We combined tables 2 and 3 in table 2 (in lines 402-403), and tables 5 and 6 in table 4(in lines 419). Also figure 3, 4, and 5 are combined in one figure named 3A,3B, and 3C in lines 453).
