# Supplementary material for: Food insecurity and water management shocks in Saudi Arabia: Bayesian VAR analysis
Source: PLoS One. 2024 Jan 24;19(1):e0296721. doi: 10.1371/journal.pone.0296721 (PMC10807787; doi:10.1371/journal.pone.0296721)
Supplement: Attachment — Submitted filename: response to reveiwers 3.docx [file pone.0296721.s003.docx]

Response to the reviewer to comments for article: **Food Insecurity and Water Management Shocks in Saudi Arabia: Bayesian VAR Analysis**

Thank you very much for your efforts and taking the time to review this manuscript. Thanks for all the suggestions and spotting points. We responded to all suggestions. Please find the detailed responses below and the corresponding revisions/corrections highlighted/in track changes and red color in the re-submitted revised (2) files.

Reviewer #3: 1. The manuscript titled “Food Insecurity and Water Management Shocks in 1 Saudi Arabia: Bayesian VAR Analysis” aims to evaluate the influence of water use efficiency as a Sustainable Development Goal on the Food Insecurity Multidimensional Index. Additionally, it estimates the impact of water efficiency on food insecurity in Saudi Arabia using the Bayesian Vector Autoregressive model. The study holds promise as a valuable contribution, but in its present form, the manuscript is too lengthy and appears to have a predominantly local focus while lacking a broader global/scientific appeal. Nonetheless, I have the following suggestions to the authors, which can enhance the manuscript:
2. The are so many typos and grammatical error in the manuscript. Please revisit.

Answer: Thanks for pointing out this. We revised the grammar errors.
3. The study is very regional. Although it will be helpful for local communities, how useful is it to the international water community?

Response: Thanks for pointing out this comment. We briefly explain the benefit of this study to the global community along the 137-142.

4. Abstract: The abstract should articulate and highlight the research problem in a broader perspective so that it is easy for the readers to easily connect to the article. The objectives should be clearly mentioned. The approach and the data used, along with the findings and their implications can be articulated subsequently. Therefore, re-writing is required in this regard. Also try to be more concise in writing without losing the bigger picture.

Response: Thank you for mentioning this. The abstract was rewritten accordingly.

5. Introduction: The introduction could benefit from a clearer statement of the research problem identified based on a more comprehensive review of the recent literature, articulation of testable hypotheses, and objectives.

Response: Thanks for mentioning this. We cited the references mentioned below to enrich the introduction.
6. Support your sentence in the introduction section with proper references such as https://doi.org/10.1016/j.ecolind.2023.110766; https://doi.org/10.3390/w15132438; <https://doi.org/10.1007/s13201-023-01928-z>.

Response: So thank for suggesting these three references. We cited all of them.

Comment: <https://doi.org/10.1016/j.ecolind.2023.110766>:

Response: Very thankful for suggesting this reference. We cited as reference no. 14

Comment: <https://doi.org/10.3390/w15132438>:

Response: Very thankful for suggesting this reference. We cited as reference no. 13

Comment: <https://doi.org/10.1007/s13201-023-01928-z>:

Response: Very thankful for suggesting this reference. We cited as reference no. 15

7. It is better to include a flowchart of the methodology.

Response: thanks for suggesting this. A flowchart was created in the methodology section in lines: 223-234.

8. The resolution of all the figures should be increased.

Response: Thanks for suggesting this. All the figure's resolutions were increased.

9. There are so many abbreviations used in the manuscript. It is suggested to insert the table of abbreviations.

Response: thanks for your comment. We already inserted the abbreviations in lines 546-575.

10. Please check that the reference should be given to all the equations used in the manuscript.

Response: All references were given for all equations.

11. Results: Please try to limit the number of tables as it will only confuse the reader. Only the most important ones can be kept in the manuscript, while additional/supporting tables and figures may be given as supplementary materials. Please maintain conciseness without neglecting the important findings. The references also need to be updated.

Response: Thanks for this comment. We reduced the tables, and we kept the important ones.

The references also need to be updated.

Response: We updated some references, e.g., 91-96, but some references about the equations (initial investors of equations) we can’t update them.

12. Conclusions: This section needs re-writing and should effectively synthesize the essential points presented in the preceding parts and make a succinct statement that reiterates the text's central message or core theme. It should leave the reader with a lasting impression and bring together the topic's overall relevance or ramifications.

Response: thanks for pointing out these points. We rewrite the conclusions accordingly.

13. Overall, the manuscript's general readability is hampered by a number of unclear/clumsily constructed sentences. Improving the clarity of language and sentence structure throughout the manuscript would enhance the readability and overall quality of the presentation.

Response: Thanks for your comment. We rechecked the manuscript accordingly.
